# Fundamental mechanism of ferroptosis: Three unanswered questions

**DOI:** 10.70401/fos.2026.0015

**Published:** 2026-01-23

**Authors:** Hanna Feinsod, Brent R. Stockwell

**Affiliations:** 1 Department of Biological Sciences, Columbia University, New York, NY 10027, USA; 2 Department of Biological Sciences, Irving Institute for Cancer Dynamics, Data Science Institute, Columbia University, New York, NY 10027, USA; 3 Department of Pathology and Cell Biology, Herbert Irving Comprehensive Cancer Center, Columbia University Digestive and Liver Disease Research Center, Vagelos College of Physicians and Surgeons, Columbia University Irving Medical Center, New York, NY 10032, USA

**Keywords:** Ferroptosis, metabolism, lipid, ROS, peroxidation, iron

## Abstract

Ferroptosis, an iron-dependent form of regulated cell death (RCD) driven by lipid peroxidation, has been extensively studied since its conceptualization in 2012 and has been suggested as a therapeutic target in many cancers and degenerative diseases. However, three fundamental questions remain unanswered about ferroptosis. First, the mechanisms by which cells execute death during ferroptosis remain elusive: The key role of lipid peroxides in triggering ferroptosis is established, but how this results in the death of a cell remains unclear. Second, the physiological role of ferroptosis throughout the human life cycle is unclear; currently, there is evidence for ferroptosis in early development, immunity, aging, and tumor suppression, but not in many other aspects of physiology. Third, and finally, the intersection between ferroptosis and other RCD modalities, such as apoptosis, necroptosis, pyroptosis, and autophagic cell death, is necessary for understanding how ferroptosis integrates into networks controlling cellular fate. Addressing these gaps in knowledge is essential for building a comprehensive understanding of this mode of cell death, as well as translating ferroptosis knowledge into effective therapeutics.

## 1. Introduction

**Figure 1.**
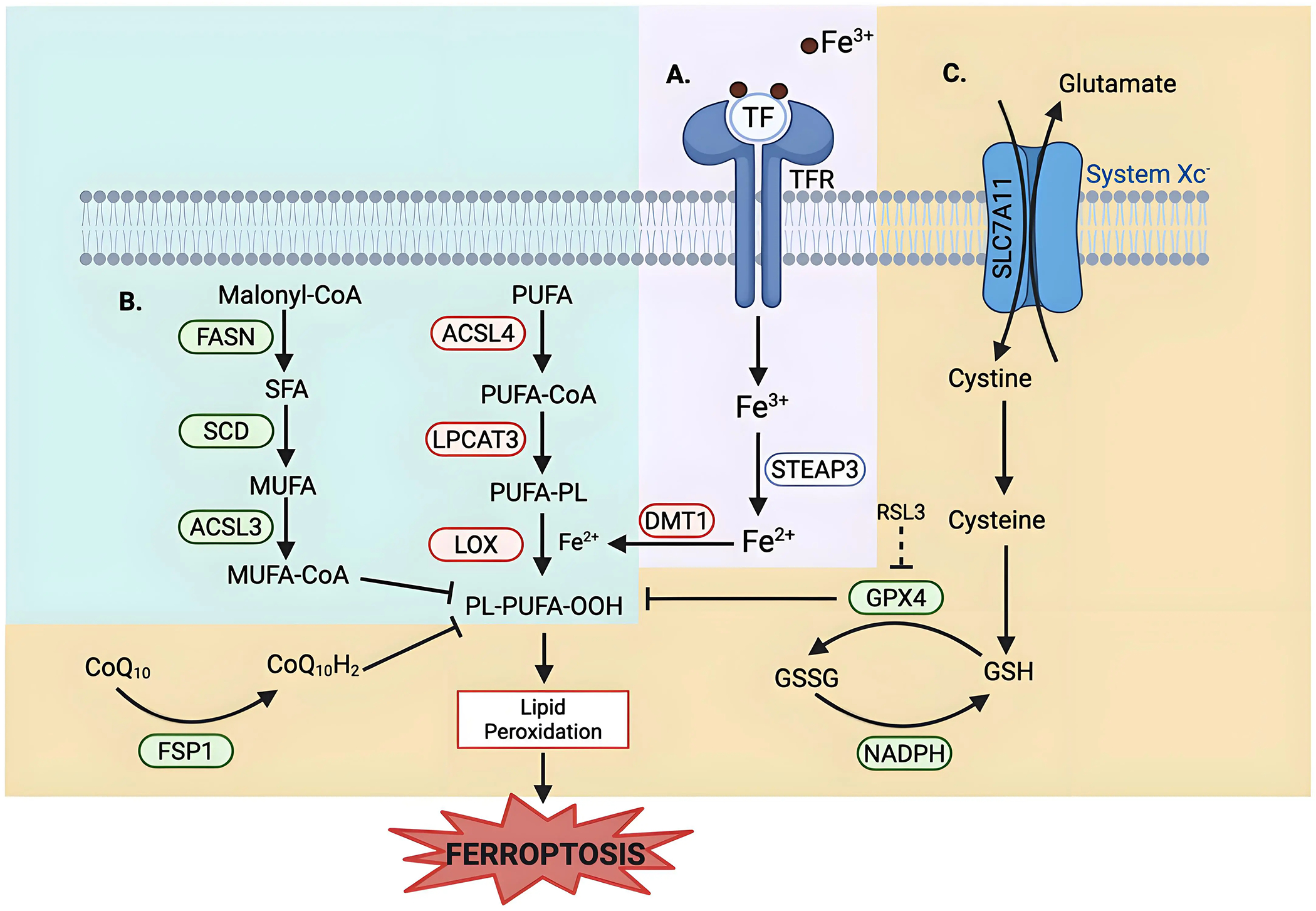
Metabolic pathways of ferroptosis regulation. Ferroptosis regulation involves (A)iron uptake and metabolism; (B) lipid metabolism, and (C) multiple antioxidant defense systems. (A) Iron Uptake and Metabolism: Iron metabolism uses TF to deliver extracellular iron (Fe^3+^) into the cells via the TFR, followed by a reduction to Fe^2+^ via STEAP3. DMT1, an iron transporter, aids in cytosolic iron import, contributing to the labile iron pool. Excess iron promotes ROS generation via the Fenton reaction, driving lipid peroxidation. (B) Lipid Metabolism: Lipid composition plays a role in regulating ferroptosis sensitivity. Enzymes that promote the synthesis and incorporation of MUFAs into phospholipids, such as FASN, SCD, and ACSL3, confer resistance to ferroptosis, as MUFAs are resistant to peroxidation. Conversely, enzymes that promote the synthesis and incorporation of PUFAs, such as ACSL4 and LPCAT3, sensitize cells to lipid peroxidation. LOX catalyzes the oxidation of PUFA-containing phospholipids, leading to the accumulation of lipid hydroperoxides. (C) Antioxidant Defense System: System Xc^−^/SLC7A11 imports cystine into the cell and glutamate out of the cells. Cystine is reduced to cysteine and is then used in GSH synthesis. GPX4, a GSH-dependent enzyme, uses reducing equivalents from NADPH to detoxify lipid hydroperoxides. RSL3 is a well-studied inhibitor of GPX4 that induces ferroptosis. An alternate defense is provided by FSP1 via reducing CoQ_10_ to block lipid peroxidation independent of GPX4. Created in BioRender.com. Red: anti-ferroptotic factors; Green: pro-ferroptotic factors; Gray: context-dependent regulator of ferroptosis; SLC7A11: solute carrier family 7 member 11; STEAP3: six-transmembrane epithelial antigen of prostate 3; DMT1: divalent metal transporter 1; FASN: fatty acid synthase; SCD: stearoyl-CoA desaturase; ACSL3: acyl-CoA synthetase long-chain family member 3; ACSL4: acyl-CoA synthetase long-chain family member 4; LPCAT3: lysophosphatidylcholine acyltransferase 3; LOX: lipoxygenase; GPX4: glutathione peroxidase 4; FSP1: ferroptosis suppressor protein 1; TF: transferrin; TFR: transferrin receptor; ROS: reactive oxygen species; MUFAs: monounsaturated fatty acids; PUFAs: polyunsaturated fatty acids; GSH: glutathione; RSL3: RAS-selective lethal 3; CoQ_10_: coenzyme Q_10_.

Ferroptosis is an iron-dependent form of non-apoptotic cell death characterized by excessive lipid peroxidation and reactive oxygen species (ROS) that result in plasma membrane rupture^[1]^. The regulation of ferroptosis is multifaceted and involves numerous pathways and biomolecules involved in metabolism, iron regulation, and redox defenses (Figure 1). The primary and most frequently studied pathway involved in ferroptosis prevention is the system Xc^−^/GSH/GPX4 axis involving Solute Carrier Family 7 Member 11 (SLC7A11), the cystine/glutamate antiporter^[2]^, glutathione (GSH), and glutathione-dependent peroxidase 4 (GPX4)^[3]^. System Xc^−^ is located in the plasma membrane and aids in the transport of cystine into, and glutamate out of, the cell via SLC7A11^[4]^. Cystine is reduced to cysteine intracellularly, which is then used in the production of GSH, which is further a cofactor for GPX4 in the reduction of lipid hydroperoxides to repair oxidative damage to lipids^[1,5–7]^. The FSP1/CoQ_10_/NAD(P)H axis is a parallel process that suppresses phospholipid peroxidation^[8,9]^. This pathway uses FSP1 (Ferroptosis Suppressor Protein 1) with its cofactor NAD(P)H to reduce coenzyme Q_10_ (CoQ_10_) (also known as ubiquinone) to CoQ_10_H_2_ (ubiquinol), which then acts as a lipophilic antioxidant^[8–10]^.

Lipid metabolism also plays a role in the regulation of ferroptosis, as peroxidation of polyunsaturated fatty acyl tails (PUFAs) in membrane lipids contributes to the triggering of ferroptosis (Figure 1)^[11,12]^. Because PUFA moieties are more susceptible to lipid peroxidation than monounsaturated fatty acyl (MUFAs) moieties, the proteins involved in the incorporation of PUFAs into membrane lipids, such as Acyl-CoA Synthetase Long-Chain Family Member 4 (ACSL4) and Lysophosphatidylcholine Acyltransferase 3 (LPCAT3), are pro-ferroptotic enzymes, while proteins involved in the incorporation of MUFAs into the membrane, such as Acyl-CoA Synthetase Long-Chain Family Member 3 (ACSL3), are anti-ferroptotic^[12–15]^. Iron metabolism also plays a key role in the regulation of ferroptosis, as iron is used in the Fenton reaction to produce ROS^[16]^. Therefore, pathways involved in iron storage, iron import and export, and iron turnover impact ferroptosis sensitivity^[17,18]^. As such, transferrin, a major iron transporter in the body, and its receptors, are important in the regulation of ferroptosis, as is CD44^[17,19]^.

Since its conceptualization in 2012^[1]^, ferroptosis has garnered significant interest, especially within oncology, with about half of ferroptosis-related publications focusing on cancer^[20]^. While research in the early days of ferroptosis focused on basic mechanisms to elucidate key genes, tools, and pathways, much of ferroptosis research has expanded to its use as a therapeutic target in cancer, neurodegenerative diseases such as Alzheimer’s and Parkinson’s disease, and ischemia–reperfusion injuries^[20–26]^. While these indications are important, a need remains for continued research into basic ferroptosis mechanisms, as gaps exist. We don’t yet understand how ferroptosis is employed in normal physiology, how cells die during ferroptosis, and how ferroptosis intersects with other forms of cell death. While these topics are fundamental, they also open the possibility for the discovery of new targets and better designed drugs for ferroptosis-related diseases.

## 2. Question One: What are the Molecular Execution Mechanisms of Ferroptosis?

Cells undergoing ferroptotic cell death have, morphologically, necrosis-like changes—the cell swells and the plasma membrane ruptures; a morphological difference from the blebbing and shrinking seen in apoptotic cells^[1,27]^. Morphological changes to mitochondria, such as fragmentation, increased membrane density, and loss of cristae, are also observed during ferroptosis^[1,28]^. Understanding how ferroptosis is executed downstream of lipid peroxidation is currently still not well defined – whether it be membrane damage directly due to lipid peroxidation, a toxic byproduct of peroxidation, lipid peroxide modulation of membrane protein activity, or a still unidentified feature^[29,30]^.

Currently, there is a suggestion that polar lipid hydroperoxides cluster together and cause membrane thinning and bending^[31]^. This may be a link to the formation of nanometer pores in the plasma membrane which have been identified as a key component in the final plasma membrane disruption^[32]^. These nanometer sized pores result in an osmotic imbalance due to the influx of water and ions. Specifically, an increase in Ca^2+^, after lipid peroxidation but prior to cell bursting, has been identified to play a key role in ferroptotic membrane degradation^[32,33]^. Additionally, truncated lipid-derived electrophile products formed after reaction with iron have been implicated as executioners of ferroptosis by damaging key proteins^[34–38]^. Moreover, the aldo-keto reductase1 (AKR1) family of aldo-keto reductases can detoxify lipid-derived electrophiles^[39]^; these genes are upregulated in ferroptosis-resistant cells and drive resistance to ferroptosis^[2,40]^.

Ninjurin-1 (NINJ1) has emerged as a multifunctional protein that is involved in mediating plasma membrane rupture (PMR) in lytic cell death pathways such as apoptosis, necrosis and pyroptosis^[41]^. The role of NINJ1 in ferroptosis is more complex and less straightforward. NINJ1 has been shown not to be relevant to the early stages of ferroptosis such as lipid peroxidation, calcium influx, or cell swelling; rather, NINJ1 was seen to regulate the late stages of ferroptosis, including membrane integrity loss, cell rupture, and damage-associated molecular pattern release after cell death^[42,43]^. Interestingly, NINJ1-deficient cells were not protected against PMR and cell death after treatment with ferroptosis inducers, but were able to delay lysis for hours after ferroptosis induction^[42,43]^. Additionally, the effect of NINJ1 was sensitive to the cell type and ferroptosis inducer used; NINJ1 deficiency has only a minor impact on cell lysis under RAS-selective lethal 3 (RSL3)-induced ferroptosis when compared to other inducers of ferroptosis^[42]^.

In addition to the specific players within the cell that allow for ferroptosis cell rounding and eventual death, understanding as to how ferroptosis is spread to neighboring cells via plasma membrane contact has recently been of interest^[44]^. Cells in which ferroptosis was induced were able to propagate lipid peroxidation and subsequent cell death to neighboring cells in a wave like pattern^[45]^. This propagation occurred upstream of cell lysis and only occurred in cells in which ferroptosis was induced via treatments that inhibit GSH and/or increase cellular iron, but not in cells in which ferroptosis was induced via direct GPX4 inhibition^[33,44,45]^.

To date, we don’t know whether morphological changes are a byproduct of how cells die and whether these changes are indicative of how ferroptotic cell death is executed. There is thus an understanding as to the importance of lipid peroxide accumulation in inducing ferroptosis, but how this results in cell death and its ability to propagate remain elusive.

## 3. Question Two: What are the Physiological Roles of Ferroptosis in Development and Aging?

The majority of the current research related to ferroptosis focuses on disease states; while important, there is still much to learn about how ferroptosis is involved in normal physiology. One area of physiological ferroptosis that has been studied is animal development. Large-scale cell death occurs in embryonic development to eliminate organs and tissues that play a role during a specific phase of development^[46,47]^. Large-scale ferroptosis during embryogenesis was first observed in the nineteenth century^[33,48]^. More recent work has explored how ferroptosis may propagate across large cell populations via trigger waves with ROS at the wavefront to aid in the shaping of limbs during development^[48]^. In both the avian and zeugopod limbs, large-scale cell death by ferroptotic waves was seen, highlighting the potential for ferroptosis to play a functional role in the muscle remodeling occurring during embryogenesis^[48]^.

Additionally, ferroptosis plays a role in the regulation of muscle fibers and the individualization of muscle limbs in muscle development via the removal of excess and improperly positioned myoblasts^[48]^. Similarly, ferroptosis has been linked to neurogenesis: The level of ROS in developing neurons must be kept within a key range, as ROS are needed in the early stages of brain development, and then suppressed by antioxidants in order to ensure stem cells mature and differentiate properly^[49,50]^. Moreover, a link between ferroptosis and erythropoiesis is seen when looking at embryonic visceral endoderm^[51]^. An increase in ferroptosis was described linking it to the differentiation and development of blood vessels^[51,52]^.

Looking at the opposite end of human life, ferroptosis has been implicated in aging. As the body ages, iron accumulates, as there is no excretion pathway for iron in men and post-menopausal women^[53]^. In multiple parts of the eye in aging individuals, an increase in iron is correlated with an increase in ferroptosis susceptibility^[54–56]^. The brain sees a similar increase in iron, and potentially ferroptosis, in the aging population^[57–58]^. This increase in iron-induced ferroptosis in elderly individuals may correlate with an increase in neurodegenerative diseases later in life^[59]^; however this may also aid in promoting tumor ferroptosis in iron-rich cancers^[60]^.

Increasing evidence has highlighted ferroptosis as an innate mechanism of malignancy suppression^[23,61–65]^. p53, a well-established tumor suppressor, uses ferroptosis via the transcriptional suppression of SLC7A11^[62]^. Similarly, cells deficient in fumarate hydratase (FH), a mitochondrial tumor suppressor, are resistant to cysteine deprivation-induced ferroptosis, highlighting that FH uses ferroptosis as a mechanism to suppress tumors^[63]^. Again, this is seen via BRCA1-associated protein 1 suppression of tumorigenesis through ferroptosis by the repression of SLC7A11^[64]^. Another occurrence of ferroptosis acting in an anti-cancer role can be seen when looking at skin cancers; specifically, Mixed Lineage Leukemia 4 deficiency, which significantly alters the epidermis, also alters the expression of key markers of ferroptosis, highlighting that ferroptosis is used here in a tumor suppressive role (and that it may play a more global role in differentiation and skin homeostasis)^[65]^. This phenomenon is seen again when looking at how membrane-bound glycerophospholipid O-acyltransferase 1 (MBOAT1) and membrane-bound glycerophospholipid O-acyltransferase 2 (MBOAT2) suppress ferroptosis by remodeling the cellular phospholipid composition^[61]^. Interestingly, MBOAT1 and MBOAT2 are transcriptionally upregulated by the estrogen receptor (ER) and androgen receptor (AR), respectively, highlighting differences in ferroptosis sensitivity seen between sexes^[61,66]^. Understanding the emphasized role of ferroptosis in cancer suppression allows for greater understanding of ways in which we can target ferroptosis to be used in anti-cancer treatments.

Although ferroptosis has been associated with embryonic development and age-related decline, its precise roles and mechanisms in development and aging remain largely unexplored. Additionally, physiological ferroptosis may play important roles at multiple stages of the lifecycle beyond tumor suppression and immunity, which have been implicated as processes depending on ferroptosis^[11,67–70]^. Understanding where, when, and how the body uses ferroptosis for normal physiology can improve our understanding of the physiological potency and regulation of ferroptosis and the ability to use it as a treatment target in disease models while simultaneously avoiding unwanted side effects in healthy tissues.

## 4. Question Three: How much Crosstalk is There Between Ferroptosis and Other Regulated Cell Death Modalities?

Regulated cell death (RCD), or programmed cell death, refers to orchestrated death by macromolecules to maintain homeostasis, which is contrasted with accidental cell death, which is an uncontrolled process of cell death triggered by injury^[71]^. Crosstalk among RCD modalities is of particular interest, as it allows for more efficient and more accurate therapeutic development. How cells decide between one death and another, how cells can switch between the different types of regulated cell death, and if specific cells have a preference for one modality over the other are questions of interest that can work to guide treatment design. Knowledge of the intersections of ferroptosis with other cell deaths is varied among RCDs. Continued research into understanding these crossroads is critical for designing combination treatments (that target two or more death modalities) to improve therapeutic outcomes.

### 4.1 Ferroptosis and apoptosis

Apoptosis is a mechanism of controlled cell death in which DNA is fragmented and membranes bleb, creating apoptotic bodies that are removed without triggering of an inflammatory response^[27,72,73]^. Apoptosis and ferroptosis share multiple regulators and upstream stress signals; however, significant differences exist in the mechanisms that induce these two types of cell death. Understanding how these cell death modalities interact and affect each other is still a priority.

**Figure 2.**
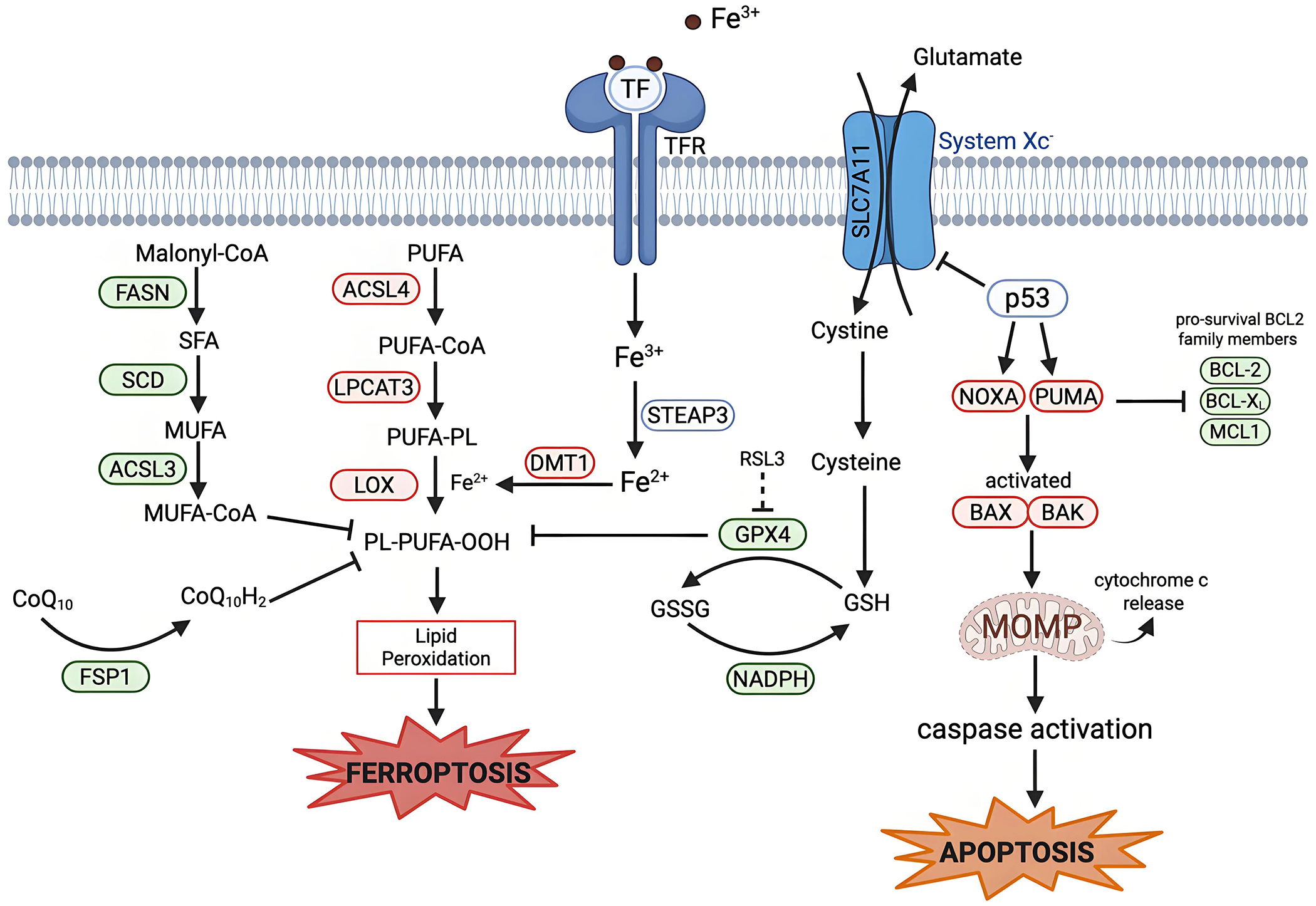
Mechanistic crosstalk between ferroptosis and apoptosis. Apoptosis is regulated by BCL-2 family proteins. The tumor suppressor p53 can transcriptionally upregulate pro-apoptotic BH3-only proteins (PUMA and NOXA). PUMA and NOXA inhibit anti-apoptotic BCL-2 family proteins (BCL-2, BCL-X_L_, and MCL1), activating BAX and BAK. BAX/BAK oligomerize to induce MOMP, which leads to release of cytochrome c and eventual caspase-9 activation and apoptotic cell death. P53 links ferroptosis and apoptosis via the suppression of SLC7A11 and induction of PUMA and NOXA. Some BCL-2 family proteins can influence both apoptosis and ferroptosis. Created in BioRender.com. Red: anti-cell death factors; Green: pro-cell death factors; Gray: context-dependent regulator of cell death; SLC7A11: solute carrier family 7 member 11; STEAP3: six-transmembrane epithelial antigen of prostate 3; DMT1: divalent metal transporter 1; FASN: fatty acid synthase; SCD: stearoyl-CoA desaturase; ACSL3: acyl-CoA synthetase long-chain family member 3; ACSL4: acyl-CoA synthetase long-chain family member 4; LPCAT3: lysophosphatidylcholine acyltransferase 3; LOX: lipoxygenase; GPX4: glutathione peroxidase 4; FSP1: ferroptosis suppressor protein 1; PUMA: p53-up-regulated modulator of apoptosis; NOXA: phorbol-12-myristate-13-acetate-induced protein; BCL-2: B-cell lymphoma 2; BCL-X_L_ B-cell lymphoma-extra-large; MCL1: myeloid cell leukemia sequence; BAX: Bcl-2-associated X protein; BAK: Bcl-2 homologous antagonist/killer; MOMP: mitochondrial outer membrane permeabilization.

In both apoptosis and ferroptosis, the mitochondria play a significant role. In apoptosis, mitochondrial outer membrane permeabilization (MOMP) is the point of no return. MOMP allows the release of cytochrome c into the cytosol, which results in caspase activation and eventual apoptotic death (Figure 2)^[74–77]^. This release is mediated by the B-cell lymphoma 2 (BCL-2) family of proteins, with specific BCL-2 family proteins such as Bcl-2-associated X protein (BAX) and Bcl-2 homologous antagonist/killer inserting into the outer membrane of the mitochondria^[78,79]^. The BCL-2 family of proteins consists of proteins that are pro-apoptotic or pro-survival; the ratio of these two types of BCL-2 family proteins determines apoptosis sensitivity in the cell^[80]^.

Some BCL-2 family proteins have been linked to ferroptotic cell death^[81]^. Abivertinib, an epidermal growth factor receptor tyrosine kinase inhibitor (TKI), was able to induce apoptosis by suppressing BCL-2 and B-cell lymphoma-extra-large and by upregulating Bcl-2 Interacting Mediator of cell death and BAX^[82]^. Abivertinib was also able to induce iron-and-ROS-induced ferroptosis in breast, cervical, and lung cancers^[83]^. Abivertinib’s ability to induce apoptosis and ferroptosis has elicited discussions on the role of BCL-2 family proteins as regulators of ferroptosis, but this has been contested, as BCL-2 family proteins can only protect and/or induce ferroptosis in some studies and not others^[83–87]^.

The perplexing role of BCL-2 family proteins in ferroptosis may be tied to the role of the mitochondria in ferroptosis. While the importance of the mitochondria in apoptosis is well documented, their role in ferroptosis is less established, but still important. When ferroptosis was first discovered, an aspect of its characterization by electron microscopy involved the shrinkage of mitochondria with increased membrane density^[1]^. The role of the mitochondria in ferroptosis, as understood today, is a metabolic one; the Tricarboxylic Acid cycle and electron transport chain produce cellular ROS and promote ferroptosis under cysteine-deprived conditions^[63]^. This highlights a distinction between these two death modalities: ferroptosis relies on active mitochondrial metabolism, whereas apoptosis is initiated by mitochondrial breakdown. When inducing ferroptosis via pharmacological inhibition or genetic elimination of GPX4, mitochondrial activity does not impact sensitivity to ferroptosis^[63]^. This suggests that there is a hierarchy of control in which GPX4 is more significant for ferroptosis induction than the mitochondria. This further highlights the question about the impact of one death modality on another. Is there a way to leverage the mitochondria so that it can aid in the induction of both RCD pathways? Mitochondria are also implicated as drivers of the formation of drug-tolerant persister (DTP) cells^[88]^: Sublethal release of cytochrome c results in a DTP cell state that is resistant to apoptosis-inducing drugs but hypersensitive to ferroptosis^[88,89]^. Lysosomal iron activation, however, can also trigger ferroptosis in drug-resistant cancer cells, as iron itself can promote cell state transitions in cancer cells^[17,90–93]^.

Other regulators of apoptosis have been connected to ferroptosis: Wildtype p53 is a tumor suppressor that is involved in the regulation of apoptosis and has been linked to ferroptosis^[94]^. p53 can trigger apoptosis through direct transcriptional activation of PUMA and NOXA, two BH3 only BCL-2 family proteins^[95,96]^. p53 has also been established to be involved in the cascade that triggers ferroptosis, mostly in a ferroptosis-promoting role, (but p53 has also inhibited ferroptosis in specific contexts)^[97]^. p53 is involved in the regulation of cellular and systemic metabolism of many of the key pathways involved in ferroptosis regulation. For example, SLC7A11, a key component involved in System Xc^−^, is a target gene that is suppressed by p53, resulting in less cystine transported into the cell (Figure 2)^[62]^. Further research is required to understand how p53 expression and mutations affect the interplay between these two forms of cell death, and whether p53 can simultaneously protect from one death modality while inducing another, or act as a switch between these two cell death processes.

Another upstream stress signal that is shared between apoptosis and ferroptosis is endoplasmic reticulum (ER) and oxidative stress. ER stress is characterized by the accumulation of unfolded or misfolded proteins in the ER, which results in the disruption of regular cellular processes^[98]^. ER stress results in the activation of the unfolded protein response (UPR) pathway, which if persistent, morphs into terminal UPR, which can promote upregulation of genes involved in iron uptake, storage, and utilization, and the inhibition of genes involved in the regulation of antioxidant defense and fatty acid metabolism, sensitizing cells to ferroptosis^[99–102]^. Similarly, when cells experience late-stage UPR, there is upregulation of pro-apoptotic genes with simultaneous downregulation of pro-survival BCL-2 family proteins. Gaining a greater understanding of how and where these two death pathways intersect can reveal targets that can be used to better induce both cell ferroptosis and apoptosis in tumors, while avoiding issues of resistance.

### 4.2 Ferroptosis and necroptosis

**Figure 3.**
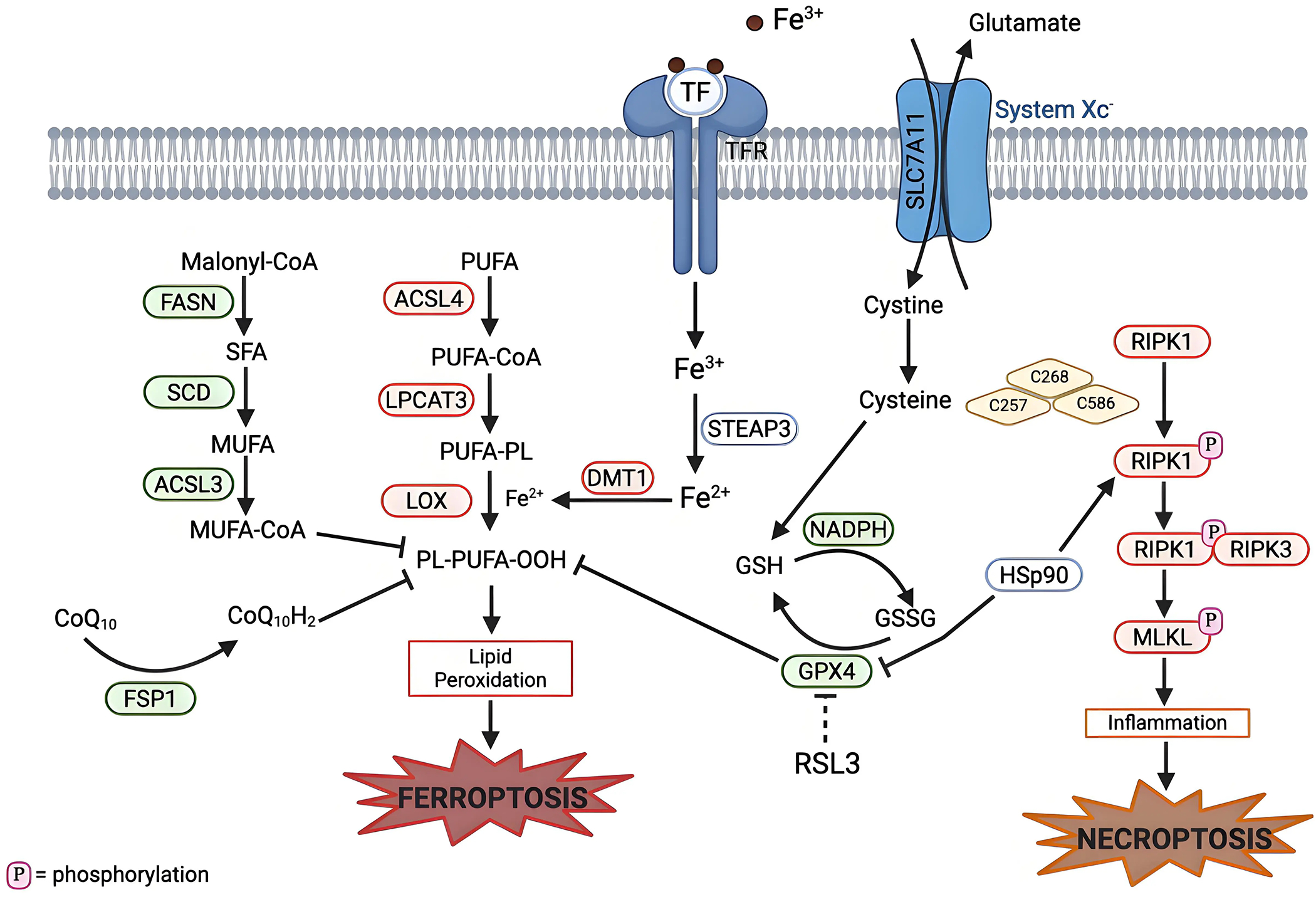
Mechanistic crosstalk between ferroptosis and necroptosis pathways. Necroptosis can be initiated by the activation of RIPK1 (in response to death receptors), which results in phosphorylation of RIPK1. Phosphorylated RIPK1 forms a complex with RIPK3 (the necrosome complex), which then phosphorylates MLKL. This leads to a pro-inflammatory response and eventual necroptotic cell death. HSP90, a molecular chaperone, links ferroptosis and necroptosis by stabilizing RIPK1 to promote necrosome assembly and also degradation of GPX4. Cysteine plays a role in both ferroptosis and apoptosis: Cysteine is involved in GSH production, which is used as a cofactor for GPX4, and also required for autophosphorylation of RIPK1. Created in BioRender.com. Red: anti-cell death factors; Green: pro-cell death factors; Gray: context dependent regulator of cell death; SLC7A11: solute carrier family 7 member 11; STEAP3: six-transmembrane epithelial antigen of prostate 3; DMT1: divalent metal transporter 1; FASN: fatty acid synthase; SCD: stearoyl-CoA desaturase; ACSL3: acyl-CoA synthetase long-chain family member 3; ACSL4: acyl-CoA synthetase long-chain family member 4; LPCAT3: lysophosphatidylcholine acyltransferase 3; LOX: lipoxygenase; GPX4: glutathione peroxidase 4; FSP1: ferroptosis suppressor protein 1; RIPK1: receptor interacting protein kinase 1; RIPK3: receptor interacting protein kinase 3; MLKL: mixed lineage kinase domain-like protein; HSp90: heat shock protein 90.

Necroptosis, like ferroptosis, is a non-apoptotic form of RCD^[103]^. Necroptosis emerged from the discovery that receptor-interacting protein kinase 1 (RIPK1) is critical for inducing regulated necrosis, while necrostatin 1 (Nec-1), a potent inhibitor of RIPK1, is able to inhibit necroptotic cell death^[104–106]^. The activation of RIPK1, receptor-interacting protein kinase 3 (RIPK3), and the tumor necrosis factor signaling pathway is critical for necroptosis (Figure 3)^[107]^.

Some studies examined the intersection between ferroptosis and necroptosis. In cells not sensitive to necroptosis, there is a time and concentration dependent hypersensitization to ferroptosis; the opposite is true as well for cells insensitive to ferroptosis^[108]^. Where this switch takes place and how these mechanisms interact with each other is unknown.

Cysteine has been highlighted as playing a key role in both these forms of cell death (Figure 3). In necroptosis, three cysteine residues (C257, C268 and C586) in RIPK1 form intermolecular disulfide bonds, which ultimately induce autophosphorylation of serine residue 161 (S161) of RIPK1. Then, the phosphorylated RIPK1 can recruit RIPK3 to form a functional necrosome^[109,110]^. In ferroptosis, cysteine aids in the production of GSH, which then acts as an antioxidant agent to protect against ferroptosis^[5]^. The use of cysteine by both these cell death pathways highlights an opportunity to exploit this sensitivity to induce both types of cell death with one treatment.

Similarly, heat shock protein 90 (HSP90) is involved in both necroptosis and ferroptosis (Figure 3). HSP90 plays a key role in the homeostasis of the RIPK1/RIPK3 complex by aiding in the stabilization and folding of RIPK3^[111]^. HSP90 is also involved in the degradation of GPX4, as it stabilizes the protein and prevents its degradation, but when HSP90 is inhibited, there is greater degradation of ferroptosis^[112–115]^. Key remaining questions involve: Where this shift from ferroptosis to necroptosis occur? How can these unique features present in both RCD modalities be targeted? Is there a way to target both types of cell death at the same time to create a multitarget treatment to increase results?

### 4.3 Ferroptosis and pyroptosis

**Figure 4.**
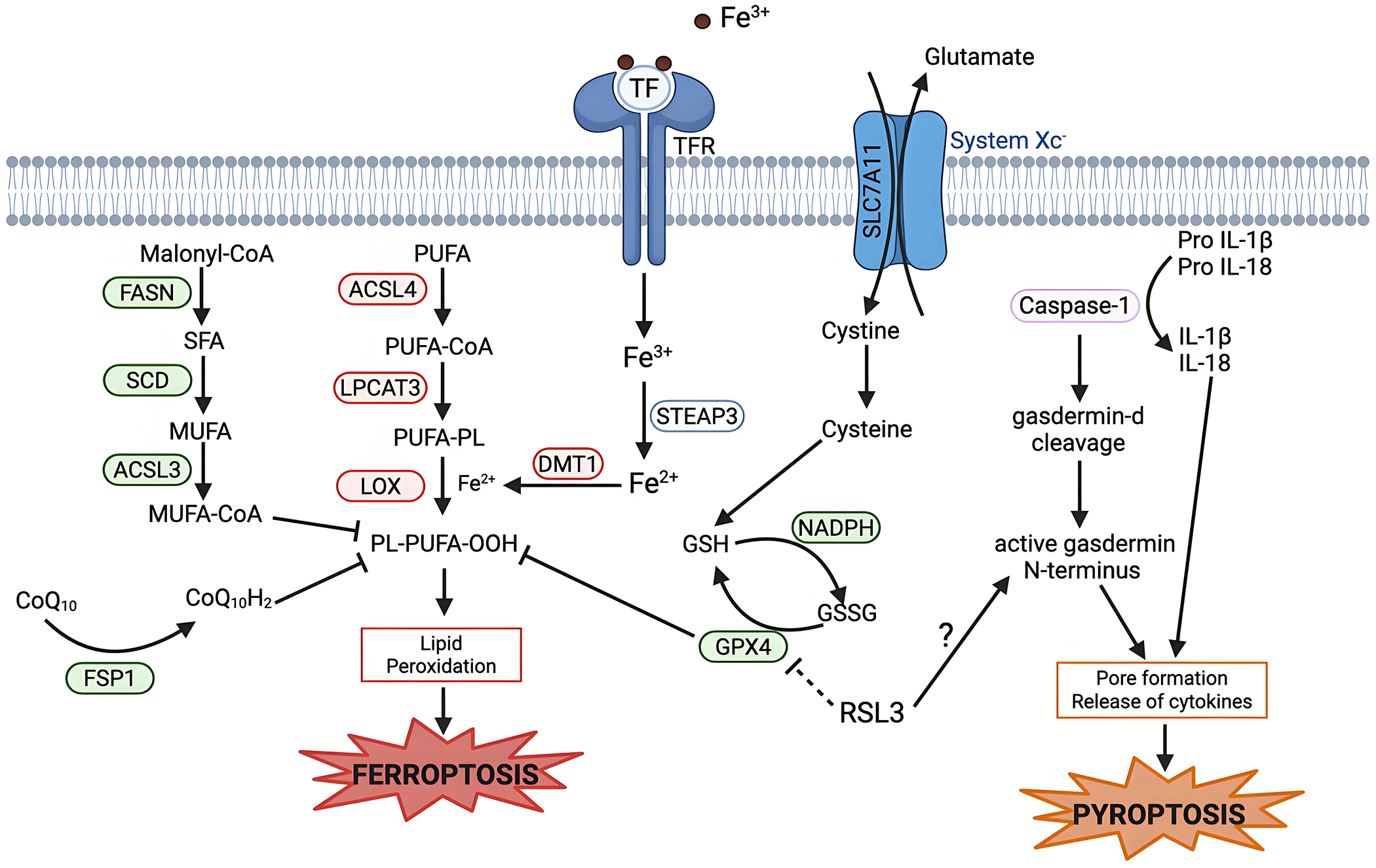
Mechanistic crosstalk between ferroptosis and pyroptosis. Pyroptosis is executed by caspase-1 activation, which leads to the cleavage of gasdermin D. The active N-terminal fragment of gasdermin D causes formation of pores in the plasma membrane and release of pro-inflammatory cytokines, leading to pyroptotic cell death. RSL3, a ferroptosis inducer, can induce pyroptosis via increasing gasdermin D cleavage in some cases. Created in BioRender.com. Red: anti-cell death factors; Green: pro-cell death factors; Gray: context dependent regulator of cell death; SLC7A11: solute carrier family 7 member 11; STEAP3: six-transmembrane epithelial antigen of prostate 3; DMT1: divalent metal transporter 1; FASN: fatty acid synthase; SCD: stearoyl-CoA desaturase; ACSL3: acyl-CoA synthetase long-chain family member 3; ACSL4: acyl-CoA synthetase long-chain family member 4; LPCAT3: lysophosphatidylcholine acyltransferase 3; LOX: lipoxygenase; GPX4: glutathione peroxidase 4; FSP1: ferroptosis suppressor protein; IL-1β: interleukin-1 beta.

Pyroptosis is an inflammatory type of cell death which involves the release of pro-inflammatory cytokines such as interleukin-1β and interleukin-18^[116]^. Pyroptosis is triggered when gasdermin proteins are cleaved into N-terminal and C-terminal fragments by proteases, usually caspases, although other proteases can also perform this function. The N-terminal fragments oligomerize to form pores in the cell membrane, which lead to swelling and eventually lysis (Figure 4)^[117–119]^.

Pyroptosis has shown overlap with other cell deaths, such as apoptosis, in which caspases play an important role^[120]^. But, the overlap with ferroptosis is less clear. RSL3, a potent ferroptosis inducer, can enhance ketamine-induced pyroptosis in the brain and induce pyroptosis in cancer cells via gasdermin cleavage (Figure 4)^[121,122]^. Similarly, with the depletion of GPX4 in myeloid cells, an increase in caspase-mediated gasdermin-D cleavage is seen^[123]^. An understanding of how GPX4 regulates both pyroptosis and ferroptosis is lacking, but the potential role of GPX4 in both suggests that lipid peroxidation may accelerate inflammasome activation and hence pyroptosis. There is much to be understood about the intersection between ferroptosis and pyroptosis. Still to be investigated are if proteins, other than GPX4, in the system Xc^−^ axis can show similar impacts on pyroptosis sensitivity as GPX4, and if this effect is bidirectional - do any of the established pyroptosis regulators affect ferroptosis sensitivity in any way?

### 4.4 Ferroptosis and autophagy

**Figure 5.**
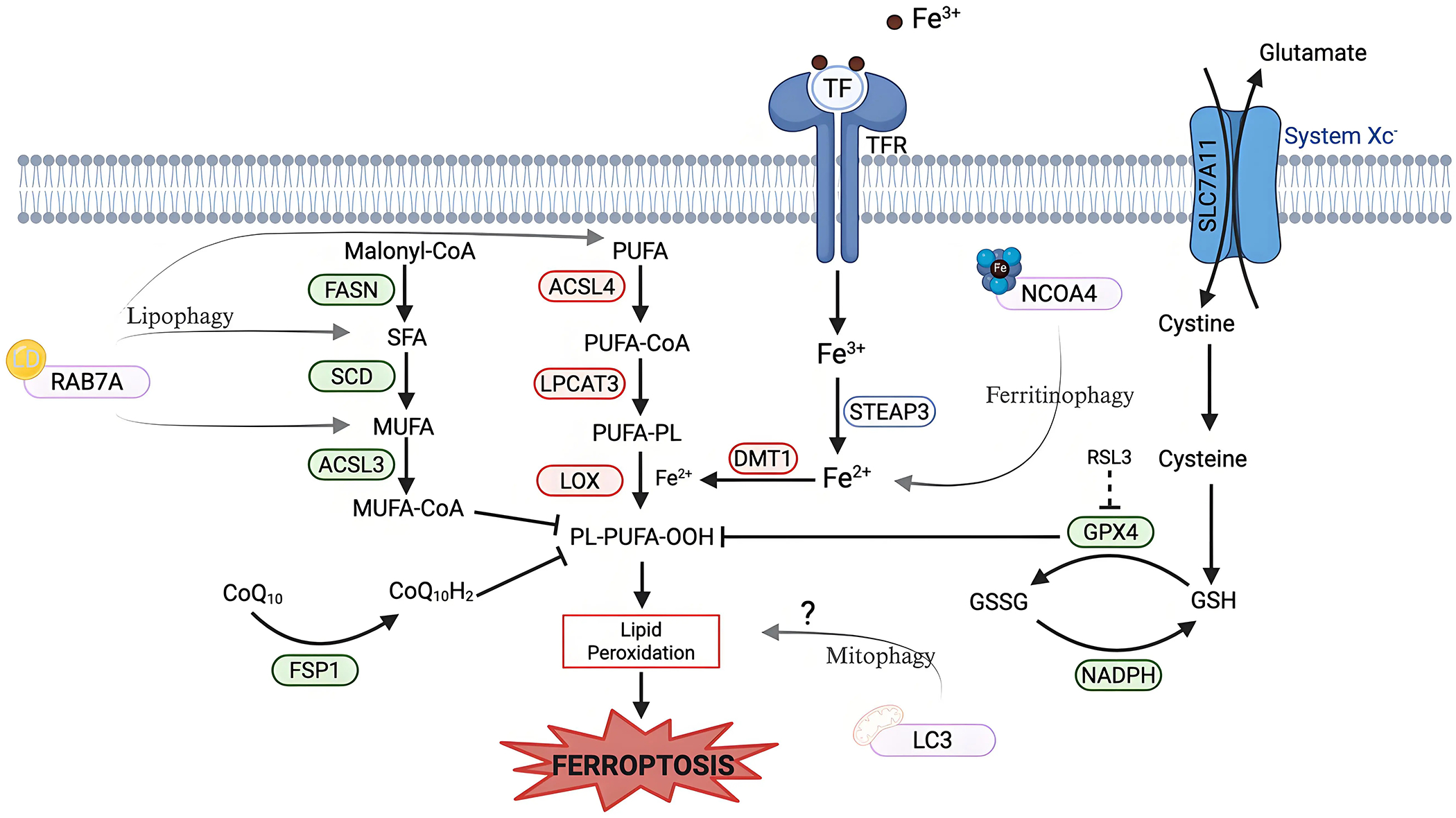
Roles of lipophagy, ferritinophagy, and mitophagy in ferroptosis regulation. Lipophagy is mediated by RAB7A, which promotes the breakdown of neutral LD into fatty acids. These fatty acids are incorporated into phospholipids that contribute to lipid peroxidation and ferroptosis sensitivity. Ferritinophagy is mediated by NCOA4, a cargo receptor that directs ferritin to the autophagosome for degradation, releasing redox-active iron into the cytosol. This increases the labile iron pool to promote ROS generation via the Fenton reaction, driving lipid peroxidation and ferroptosis. Mitophagy requires binding to LC3 on the autophagosome. The role of mitophagy in ferroptosis is not well defined, as mitophagy can both trigger ROS and reduce mitochondrial ROS, while reducing GPX4. Created in BioRender.com. Red: anti-cell death factors; Green: pro-cell death factors; Gray: context dependent regulator of cell death; SLC7A11: solute carrier family 7 member 11; STEAP3: six-transmembrane epithelial antigen of prostate 3; DMT1: divalent metal transporter 1; FASN: fatty acid synthase; SCD: stearoyl-CoA desaturase; ACSL3: acyl-CoA synthetase long-chain family member 3; ACSL4: acyl-CoA synthetase long-chain family member 4; LPCAT3: lysophosphatidylcholine acyltransferase 3; LOX: lipoxygenase; FSP1: ferroptosis suppressor protein; NCOA4: nuclear receptor coactivator 4; LC3: microtubule-associated protein 1 light chain 3; RAB7A: ras-related protein Rab-7a; LD: lipid droplets; ROS: reactive oxygen species; GPX4: glutathione-dependent peroxidase 4.

Autophagy is a cellular lysosomal process in which the cell degrades and recycles biomolecules, proteins, and organelles to maintain intracellular homeostasis^[124–126]^. These macromolecules are degraded by the lysosome into raw materials as a mechanism for adaptation to starvation or stress^[124,127]^. Autophagy plays an important role in the regulation of ferroptosis and, in most contexts, promotes ferroptosis (Figure 5)^[112,128]^. Ferritinophagy is a type of autophagy that regulates the distribution and utilization of iron ions in cells^[128]^. Ferritin, the primary intracellular iron storage protein, undergoes degradation via autophagy, leading to the release of iron from the lysosome, resulting in elevated cytosolic labile iron, facilitating ferroptosis^[128–131]^. Additionally, lipophagy, the degradation of lipid droplets, results in increased lipid release, leading to lipid peroxidation and ferroptosis^[132]^. Mitophagy, the selective autophagy of mitochondria, has a more complicated relationship to ferroptosis^[133]^. Mitophagy can be triggered by ROS but can also reduce mitochondrial ROS, protecting from ferroptosis^[134]^, and GPX4 can be recruited to the mitochondria in the activation of mitophagy, which reduces free GPX4 and can promote ferroptosis^[135,136]^.

Thus, autophagy mainly aids in the induction and sensitization to ferroptosis, but mitophagy has a more complicated impact on ferroptosis. A more detailed understanding of how mitophagy regulates mitochondrial ROS and ferroptosis in different contexts is needed.

## 5. Perspectives and Conclusions

In the years since ferroptosis was conceptualized, it has emerged as a distinct and biologically significant form of regulated cell death, with clear relevance to cancer, neurodegeneration and other disease states^[20–26,137–139]^. Despite substantial progress in defining its molecular regulators and biochemical hallmarks, fundamental gaps remain in our understanding of how ferroptosis is executed, when and where it functions physiologically, and how it interfaces with other cell death programs. Addressing these questions is essential for the integration of ferroptosis into the framework of cellular fate control and to fully reap its therapeutic potential.

A major priority for future research is to elucidate the final steps and execution of ferroptotic death. While lipid peroxidation and osmotic flux are defining features of ferroptosis the downstream events that result in cellular demise are still undetermined. Future work can look at how the membrane nanopores are formed, if there are specific sites within the membrane that are more susceptible to this pore formation, and how that correlates to the fatty acid content of the membrane. Additionally, determining any biophysical thresholds that trigger ferroptosis can allow for a greater understanding of the critical execution steps of ferroptosis.

Equally important is defining the physiological roles of ferroptosis across tissues and developmental stages. While emerging work highlights the function of ferroptosis in embryogenesis, aging, and tumor suppression, there remains a continued need to map where ferroptosis occurs under normal physiological conditions. Identifying cell-type specific vulnerabilities to ferroptosis and distinguishing physiological from pathological activation are possible next steps in mapping the physiological roles of ferroptosis.

In addition, crosstalk between ferroptosis and other forms of regulated cell death can be leveraged to maximize drug treatments. Ferroptosis interacts with apoptosis, necroptosis, pyroptosis, and autophagy regulation pathways through shared signaling, dependencies, and stress responses. Dissecting the interplay of these pathways can illuminate more of the cell fate scaffolding and can reveal opportunities for combination therapeutic strategies which can target more than one pathway at a time.

## Data Availability

Not applicable.
